# Spontaneous Bacterial Empyema in Liver Cirrhosis: An Underdiagnosed Pleural Complication

**DOI:** 10.4103/1319-3767.37809

**Published:** 2008-01

**Authors:** Naglaa A. H. Allam

**Affiliations:** Lecturer of Hepatology, National Liver Institute, Menofeya University, Egypt

**Keywords:** Cirrhosis, hepatic hydrothorax, spontaneous empyema

## Abstract

Spontaneous bacterial empyema, defined as spontaneous infection of the pleural fluid, represents a distinct complication of hepatic hydrothorax with a different pathogenesis, clinical course and treatment strategy from those of empyema secondary to pneumonia. Nearly 40% of episodes of spontaneous empyema are not associated with spontaneous bacterial peritonitis (SBP) or even ascites. The condition portends a poor prognosis, and is frequently under-diagnosed. This article reviews the pathogenesis, diagnosis and management of spontaneous bacterial empyema.

Hepatic hydrothorax is defined as a significant pleural effusion, usually greater than 500 ml, in a cirrhotic patient without any underlying pulmonary or cardiac diseases. It appears to be a relatively uncommon complication of portal hypertension with an estimated prevalence of 5–12% in patients with the cirrhosis of the liver. Hepatic hydrothorax is usually right-sided (65–87% of reported cases) but may be left-sided or bilateral.[1]

Spontaneous bacterial empyema, defined as the spontaneous infection of the pleural fluid, represents a distinct complication of hepatic hydrothorax. This term may be confusing because in most cases there is no evidence of pus or abscess in the thoracic cavity and indeed, the pathogenesis, clinical course and treatment strategy of spontaneous bacterial empyema (SBEM) are different from those of empyema secondary to pneumonia. Hence, some authors have called it as spontaneous bacterial pleuritis. However, this name has not gained acceptance and most published studies referring to infections of the pleural fluid in cirrhotics use the term SBEM.[2] Spontaneous bacterial empyema was reported to be present in as many as 13% of the hospitalized cases of hepatic hydrothorax and some authors reported even a higher incidence reaching 30%[3] comparable to spontaneous bacterial peritonitis (SBP). SBEM is associated with a deteriorating prognosis with an estimated mortality rate over 20%.[1]

The aim of this review is to highlight the pathogenesis and diagnostic features of spontaneous bacterial empyema.

## PATHOGENESIS OF SBEM

The pathogenesis of SBEM remains unclear. It might occur as a result of a direct bacterial spread from the peritoneal cavity. However, it was reported that nearly 40% of the SBEM episodes were not associated with SBP. Moreover, SBEM may occur even in the absence of ascites. In such cases, a transient bacteremia that infects the pleural space could be the underlying pathogenetic mechanism. Since patients who develop SBP have low ascites complement levels and low ascitic fluid opsonic activity, reflecting the importance of local factors in the prevention of the colonization of ascitic fluid by pathogenic bacteria, likewise defective local pleural factors were speculated to enhance infection in these patients. The impaired opsonic activity of the pleural fluid was found to enhance bacterial translocation.[4] Sese *et al*., showed that in addition to lower pleural fluid opsonic activity, patients who develop SBEM also have lower levels of C3 than those who do not develop SBEM. The causative micro-organisms in most cases of SBEM are *Escheria coli*, *Streptococcus* species, *Enterococcus* and *Klebsiella*.[5]

The risk factors for the development of SBEM in patients with cirrhosis have been addressed in studies and are shown in Table 1. A high Child-Pugh score, decreased pleural fluid total protein and low levels of C3 component in pleural fluid were proved risk factors for the development of SBEM. In addition, SBP is a predictive factor of SBEM.[6] Because there is a very good correlation between pleural C3 and total protein levels, Sese *et al*. recommended the use of pleural fluid total protein concentration in clinical practice to detect those patients at a risk for developing SBEM.[5]

**Table 1:** Risk factors for development of spontaneous bacterial empyema

High Child-Pugh score
Low serum albumin
Low pleural fluid protein
Low pleural fluid C3
Spontaneous bacterial peritonitis

## DIAGNOSIS OF SBEM

The infection of the pleural fluid is frequently associated with few localizing signs. Therefore, a high index of suspicion is essential for the diagnosis of SBEM. Diagnostic criteria are demonstrated in Table 2. Any patient with hydrothorax who develops fever, pleuritic pain, encephalopathy or unexplained deterioration in renal function should undergo diagnostic thoracocentesis.[6] SBEM is defined as pleural fluid with a polymorphonuclear (PMN) cell count > 500 cells/mm^3^ or positive culture with PMN cell count > 250 cells/mm^3^ with the exclusion of a parapneumonic effusion. In fact, SBEM is rarely diagnosed, because thoracocentesis is not routinely performed in cirrhotic patients with hepatic hydrothorax. Furthermore, since SBEM is probably an infection that involves a low concentration of bacteria as is SBP, conventional cultures are not sufficiently sensitive to diagnose the condition. Pleural fluid culture should be performed by inoculating 10 ml pleural fluid into a TSB blood culture bottle at bedside since it contains an opsonin inhibitor that protects bacteria from further complement- or phagocyte-mediated killing.[2]

**Table 2:** Diagnostic criteria of spontaneous bacterial empyema

Serum/pleural fluid albumin gradient >1.1 g/dL
Polymorphonuclear leukocyte count >500/mm^3^ or positive fluid culture with PMN cell count > 250 cells/mm^3^
Absence of pneumonia or a contiguous infection process on chest radiography

Routine pleural fluid analysis showed limited diagnostic efficacy in the diagnosis of SBEM since lactate dehydrogenase, total protein and glucose were not reported to differ significantly between the patients with SBEM and those with noninfected effusion and it did not correlate with PMN cell count. Hence, the diagnosis of SBE should not be overlooked when these parameters are found within the expected levels found in the transudate.

Castellote *et al*. showed that the analysis of pleural fluid with a reagent strip for leukocyte esterase might represent a rapid, easy-to-use and inexpensive tool for the diagnosis of SBEM in cirrhotic patients. However, more studies are required to confirm these results.[7]

## MANAGEMENT

In patients who develop SBEM, therapy with an intravenous third generation cephalosporin antibiotic such as ceftriaxone (1 g every 24 h for 7–10 days) should be commenced immediately after the diagnosis is made. Given that the patients who develop SBEM have approximately a 20% mortality during therapy and that a beneficial effect on mortality has been demonstrated with albumin infusion in the setting of SBP, some authors also use albumin therapy at 1.5 g/kg on day 1 and 1.0 g/kg on day 3 in the setting of SBEM, although albumin infusion has not been specifically studied in the setting of hepatic hydrothorax and SBEM. In cases where there is slow clinical recovery, a repeat thoracentesis is recommended to document that the patient is responding to treatment. Because its insertion in cirrhotic patients can be harmful, a chest tube should not be used in the treatment of SBEM.[6]

The main goal of treatment is relief of symptoms and control of infection until liver transplantation can be performed. Recently, Xiol *et al*. studied the outcome of liver transplantation in patients with hepatic hydrothorax and showed that long-term evolution was similar between patients with refractory hepatic hydrothorax or spontaneous bacterial empyema and those with noncomplicated hepatic hydrothorax. Therefore, liver transplantation might be an excellent therapeutic option for patients with hepatic hydrothorax even when complicated by empyema.[8]

In conclusion, SBEM is a frequent but underdiagnosed complication of hepatic hydrothorax and portends a poor prognosis. More studies are required to elucidate the underlying pathogenetic mechanism and the natural course of SBEM. Meanwhile, its possible occurrence should be borne in mind in cases of hepatic hydrothorax who develop fever, encephalopathy or unexplained deterioration of renal functions, particularly if they have high Child-Pugh score with or even without SBP. A diagnostic thoracentesis with subsequent culture of pleural fluid should be performed in these patients.
